# Cross-border reproductive healthcare attitudes and behaviours among women living in Florence, Italy

**DOI:** 10.1186/s12913-022-07621-2

**Published:** 2022-02-22

**Authors:** Stephanie Meier, Jaziel Ramos-Ortiz, Kelsie Basille, Alyson C. D’Eramo, Adria M. Diaconu, Lesley J. Flores, Savannah Hottle, Kaylee Mason-Yeary, Yumary Ruiz, Andrea L. DeMaria

**Affiliations:** 1 Division of Consumer Science, Purdue University, West Lafayette, IN USA; 2 School of Nursing, Purdue University, West Lafayette, IN USA; 3 Department of Health & Kinesiology, Purdue University, West Lafayette, IN USA; 4 Department of Nutrition Science, Purdue University, West Lafayette, IN USA; 5 School of Health Sciences, Purdue University, West Lafayette, IN USA; 6 College of Education, Purdue University, West Lafayette, IN USA; 7 Department of Public Health, Purdue University, 812 West State Street, West Lafayette, IN 47907 USA

**Keywords:** Italy, Qualitative Methods, Cross-Border Reproductive Care, Reproductive Tourism, Medically Assisted Reproduction, Infertility, Social Norms

## Abstract

**Background:**

The number of women living in Italy and seeking cross-border reproductive care (CBRC), especially for medically assisted reproduction (MAR), has increased. The purpose of this study was to explore CBRC attitudes and behaviours among a cohort of reproductive-aged women who have never engaged in CBRC to gauge social and cultural perceptions and gain a deeper understanding of family planning discourse.

**Methods:**

In-depth interviews were conducted during May – June 2018 with 30 women aged 18–50 living in or around Florence, Italy and enrolled in the Italian healthcare system. Interviews offered in-depth insight into CBRC attitudes, behaviours, and experiences among a cohort of women living in Italy who had never engaged in CBRC. Researchers used an expanded grounded theory through open and axial coding. Emergent themes were identified via a constant comparison approach.

**Results:**

Three themes and two subthemes emerged from the data. Participants discussed how limitations in Italy’s access to MAR can lead women to seek reproductive healthcare in other countries. Women had mixed feelings about the effect of religion on legislation and reproductive healthcare access, with many views tied to religious and spiritual norms impacting MAR treatment-seeking in-country and across borders. Participants perceived infertility and CBRC-seeking as socially isolating, as the motherhood identity was highly revered. The financial cost of traveling for CBRC limited access and exacerbated emotional impacts.

**Conclusions:**

Findings offered insight into CBRC perceptions and intentions, presenting a deeper understanding of the existing family planning discourse among reproductive-aged women. This may allow policymakers and practitioners to address social and cultural perceptions, increase access to safe and effective local care, and empower women in their family planning decisions.

## Introduction

In 2004, the Italian parliament passed Law 40, which placed restrictions on medically assisted reproduction (MAR), or any form of non-coital conception [1, 2], and was met with much criticism [3–7]. The law has since been repealed due to this considerable backlash, resulting in Italian Constitutional Court judgements that guaranteed more people the right to access MAR and make decisions about their family planning futures [2]. While these changes enhanced access, barriers persist among individuals and couples seeking MAR, such as number of embryos implanted via in vitro fertilisation (IVF), ability to store and freeze embryos, and who is eligible (single women, lesbian and gay couples, and non-cohabitating couples are currently excluded, as well as people of certain ages) [2, 3, 8]. As a result, few Italians and people living/receiving healthcare in Italy can receive the MAR support they need, resulting in travel to other countries to obtain the desired healthcare and assistance [2, 9].

Cross-border reproductive care (CBRC), sometimes referred to as reproductive tourism [10, 11], is rapidly growing in Europe [12]. Italy has among the lowest fertility rates in Europe, which has resulted in high frequency of CBRC-seeking [12, 13]. Shenfield et al. [14] found the majority of Italians reported seeking services in Spain and Switzerland and most (70%) did so due to legislation limitations and quality of care available [14]. Italian travellers were most often referred to MAR-friendly countries, such as Spain with more relaxed laws [12], by their physicians (55.2%), friends (25.8%), and the Internet (25.3%) [14].

Research suggests infertile individuals, particularly women, may suffer from stigma attached to being childless, which can reduce overall quality of life [15, 16]. This may be particularly heightened in Italy as the culture places much importance on motherhood and childbearing [17–20]. Studies have found that women who perceive that others view them as damaged or incomplete due to their infertility are more likely to suffer from poor mental health [16, 21]. The guilt and shame felt by infertile individuals may also impact their healthcare-seeking behaviours [20, 22]. Infertility treatments are included in the public system in Italy across 300 centres (71,686 cycles performed in 2017) [23–25]; however, because these are discretionary healthcare expenditures, women may experience shaming or stigma if they choose to use the public system, rather than pay for treatments out-of-pocket through the private system [26]. This may impact their overall experience of accessing care in an already emotionally fraught situation [27].

Prior literature [12, 28] suggests negative MAR perceptions in Italy relate to concerns about contradicting religious determinism to achieve desired family size. Historically, the Catholic Church has morally opposed MAR, as it separates procreation and sexual function [29, 30], by fixating on “natural law” [31]. Law 40 protects and reinforces this idea of the “acceptable” family unit, which is composed of biological children with heterosexual parents who are either married or stably cohabitating [2, 32]. This may pose a threat to the reproductive freedom and autonomy of women and couples in Italy related to seeking infertility assistance [33]. In Italy, 80% of residents indicate affiliation with Catholicism [34], suggesting further exploration of the role religion plays in MAR, family planning, and CBRC.

### Study Purpose

The purpose of this study was to explore CBRC attitudes, behaviours, and intentions among a cohort of reproductive-aged women who have never engaged in CBRC. Using a cohort who has never engaged in the behaviour may offer insight into CBRC perceptions and intentions, presenting a deeper understanding of the existing family planning discourse among reproductive-aged women. Findings may provide context to how women who have not yet engaged in CBRC feel about it, perceived barriers and facilitators to reproductive health services in Italy, and the need to employ women’s voices and social perceptions in campaigns and policy development.

## Methods

As part of a larger women’s health study [35, 36], researchers conducted 30 English-language interviews with reproductive-aged women (18–50 years) living in or near Florence, Italy, who were proficient in conversational English and had a history of seeking healthcare services in Italy. The interviews were held between May and June 2018 in Florence, Italy. Recruitment efforts included flyers written in English and Italian distributed throughout the city centre on community boards, in universities and libraries, and local businesses (e.g. pharmacies, launderettes, restaurants, cafes). Recruitment also included social media posts in various community pages that could be easily shared, and in-person provision of flyers and study information if women expressed interest in learning more about the study. Participants were also asked to refer other eligible women to participate (i.e. snowball sampling), to enhance the study pool [37]. Participants completed anonymous demographic surveys following each interview. The study was approved by the Purdue University Institutional Ethics Review Board, with a letter of support from the partnering Italian university, Florence University of the Arts. The research conformed to all ethical principles for medical research on human subjects, per the Declaration of Helsinki.

### Interviews

Interviews were conducted at convenient times and locations for participants and researchers. Each interviewer obtained written informed consent before beginning the interview process, including for audio-recording. Interviews lasted approximately one hour and participants received a €25 gift card as compensation. The interviews were audio-recorded.

The interview followed a semi-structured protocol, allowing flexibility for interviewers to add, change, or reorder questions and for participants to introduce novel concepts [37, 38]. Each interview began with questions about participants’ daily lives to increase rapport [37]. As the interview progressed, questions explored family planning and healthcare (e.g., “Where do you typically go to receive women’s health care (e.g., gynaecology services)?” and “Do other people’s opinions (e.g., friends, family members, partners, children) affect your women’s health decisions? Can you tell me more about that?”), infertility knowledge (e.g., “Have you or someone you know ever experienced infertility?”), perceptions of medical and social support (e.g., “Can you describe the role of the healthcare system in infertility treatment?; “How do women seek support from partners related to infertility?”), cultural attitudes towards infertility (e.g., “How is infertility viewed by Italian society?”), CBRC experiences (e.g., “Is it common for Italian women to travel outside of Italy to receive infertility or other reproductive health services?”), and perceptions of reproductive limitations in Italy (e.g., “What limitations do the Italian healthcare system have related to reproductive technologies?”). This range of questions provided a robust understanding of Italian women’s perspectives. Interviews continued until data reached theoretical saturation, with study concepts fully developed. Data were collected and transcribed verbatim by 16 undergraduate and one graduate student participating in a research-based study abroad programme offered by Purdue University, West Lafayette, IN, USA, which is why all interviews were conducted in English. All students were trained in graduate-level qualitative research methodologies and immersed in the Florence community for two months. Two graduate students (first and second author) and five undergraduate students (third through eighth authors) completed the data coding and analyses, which was coordinated by the study’s principal investigator (last author).

### Data analysis

Grounded theory offered a conceptual lens to understand the data, privileging participant experiences [39]. Participant words, phrases, and experiences provided in vivo codes throughout coding, consistent with grounded theory [39]. The authors first conducted immersive content review to ensure familiarity with all data transcripts [40] and noted immerging patterns and ideas for codes and themes [40]. Following familiarization, researchers utilized a deductive/inductive approach for codebook development to allow greater representation of the data during the coding process [41, 42]. Initial codes were generated deductively and compiled into a preliminary codebook draft [42]. The inductive component permitted researchers to modify or add codes to capture emerging themes [42]. HyperRESEARCH 4.5.1 was used for transcript storage, data management, and coding [43]. Authors completed multiple rounds of coding until saturation was reached (i.e., no new codes were added) [40]. Coding was primarily performed by authors three through nine, with frequent discussions and guidance from authors one and two, and the last author (the primary investigator).

Following the coding process, data were collated into draft themes and subthemes. Theme development was data-driven and closely reflected participant responses [44]. Resulting themes and subthemes were reviewed and approved by all authors and further refined and defined through drafting [40]. Researchers thoroughly and collaboratively discussed and analysed individual themes and incorporated relevant subthemes to provide structure and differentiate levels of meaning [40]. Any discrepancies were resolved via consensus until final themes were fully agreed upon by all.

## Results

Interviews offered in-depth insight into CBRC attitudes, behaviours, and experiences among a cohort of women living in Italy who had never engaged in CBRC. See Table 1 for participant characteristics. Three primary themes and two subthemes are presented below with representative quotes.

**Table 1:** Participant Characteristics

	***n* = 30**
**Age** (in years)	35.0 ± 7.6
**Marital Status**	
Single	11 (36.7%)
In a relationship	9 (30.0%)
Married	10 (33.3%)
**Sexuality**	
Straight / heterosexual	29 (96.7%)
Bisexual	1 (3.3%)
**Children**	
At least one child	8 (26.7%)
Pregnant	1 (3.3%)
No children	21 (70.0%)
**Education**	
Completed or initiated university	11 (36.7%)
Completed or initiated post-graduate studies	18 (60.0%)
**Primary Healthcare**	
Public system	10 (33.3%)
Private system	9 (30.0%)
Both public and private interchangeably	11 (36.7%)
**Country of Origin**	
Italy	18 (60.0%)
Some Other Country (i.e., Germany, France, United States)	12 (40.0%)
**Time Lived in Italy** (in years)	19.1 ± 14.8
**City of Residence**	
Florence	29 (96.7%)
Other Tuscan City	1 (3.3%)

Numbers presented as *n*(%) or Mean ± Standard Deviation. Numbers that do not add to 100% reflect missing data

### CBRC: ‘It’s Important to Have a Chance to Try’

Participants discussed how limitations in Italy’s access to MAR can lead women to seek reproductive healthcare in other countries, including 10 participants who knew individuals who had sought MAR abroad. Participants highlighted Italy’s policy of restricting IVF to three-rounds and constraints on freezing sperm as one reason to travel for care. One-third of participants (*n* = 10) knew individuals who had sought MAR abroad.

“There are [barriers] in Italy [...] the laws [are] very specific, you can’t do many things that you are able to do in other countries. If you don’t want to accept the fact that it is very difficult to have children, then you can’t do much more unless you go abroad.”

“I have to say that in Italy now we do have legislation on [IVF] that is very restrictive. We are no [longer] allowed to make many [IVF] attempts and we cannot freeze the sperm for further attempts…maybe some couples might decide to go [abroad] to do it or [attempt] something that hasn’t been approved yet.”

“A friend of mine [went] abroad and this is what I’m telling you…it’s too complicated [in Italy]. Maybe it’s possible. Abroad it’s very easier,’ and another participant noted ‘a friend of mine has been trying for many years, she didn’t get any child so she [travelled] to see what could be done to have children. And she [had] luck, she had twins.”

Participants viewed Spain and Switzerland as the most common European countries for Italian couples to seek CBRC. findings suggest that women perceive barriers related to accessing advanced MAR in Italy and viewed these as likely influencing couples or individuals to travel to other European countries, like Spain and Switzerland, to seek care.

“It is becoming a new business for Spain because they allow more attempts. Even if it’s very stressful for couples who really want to have a baby, it’s important to have a chance to try.”

“I know…some person go to Switzerland for trying to have a baby without man.”

### Religion: ‘Let’s not forget that it’s still a Catholic country’

Participants had mixed feelings about the effect of religion on legislation and reproductive healthcare access and discussed what regions of Italy they thought had the most experience. Most participants, however, recognized the presence of religion in Italian policy related to infertility and MAR, even if this occurred. Some participants were opposed to influencing policy and law and suggested that norms surrounding what is and is not acceptable for MAR treatments stemmed from broader religious sentiments. A few participants described an interplay of fatalism and infertility acceptance, including MAR treatment-seeking. Overall, deterministic views tied to religious and spiritual norms may impact acceptability of infertility treatment-seeking in-country and outside.

“Religion is one of the biggest barriers we have in this kind of thing... having the Vatican right here it’s making a big wall around all these kinds of things and it will take many years to bring it down.”

“Let’s not forget that it’s still a Catholic country and even though it’s supposed to be a line with a non-religious state, the Church has a lot of say in it and I think sometimes it’s harder than it should be for women to get the treatment that they wanted.”

“There was a referendum many years ago that decided for a more restrictive law in this kind of [MAR] procedures and they think the psychological barrier is that we are Catholics. And so, there is a religious [component].”

“[The] Vatican is like that power that is just like sitting there all the time and its always… always interfering with the laws...the thing about infertility here is that...it’s also because of the Catholic religion and certain policies there’s only so far you can go with... IVF and things like that. I think they...I don’t think they give you as many chances as they do in other countries you know.”

“Maybe they were a little bit embarrassed you know again it’s [infertility] here, that you have this, Catholic church on our shoulder that says oh you can’t do this.”

### Societal Norms: ‘There is so much pressure on women’

#### Motherhood identity

Participants often discussed the societal pressure placed on women surrounding reproduction, including to become mothers to satisfy cultural norms. The motherhood identity appeared to overpower other identity roles, resulting in especially debilitating social consequences among women experiencing infertility. Women were often blamed for reproductive health complications, placing an additional burden when the expected motherhood role could not be achieved. Achieving the motherhood identity norm imposed stress upon Italian women, which may further impact their reproductive health futures.

“In Italy, the family is very important…it is the base of society… so having a child is, of course, a big value for a family, it’s considered normal. If you can’t get children, for sure there is a kind of sadness around you.”

“When there is [infertility] in a couple they usually think that it is the woman’s fault [because] women are expected to have kids. For example, I am 28 and people have started saying ‘Oh when are you going to get married?’ ‘When are you going to have kids?’ ‘You’re running out of time.’ So, let’s say if I am 35 and I cannot get kids because I have infertility problems. People will start saying, ‘Oh you’re too late, your cousin had kids at 25, so you waited too long.’”

#### Healthcare provider roles

Participants described healthcare providers as supportive of women experiencing reproduction complications, including creating opportunities for open communication to facilitate support, when needed. These narratives highlighted the assistance providers offer to reach women’s reproduction goals. Compassionate healthcare providers offered wraparound treatment services to address the physical and psychological impacts of reproductive health complications. However, some described cases where providers may not have encouraged women’s goals and refused treatments based on personal beliefs, hindering access to services.

“At this fertility clinic, before you go through IVF, you have to have counselling. To make sure that you’re ready for it.”

“Sometimes you find [a provider who doesn’t agree], not for infertility but for certain kinds of treatments or the level of treatment that you request [for infertility]. And if it’s hormones it’s fine but if you want to do [IVF]…it’s expensive…and couple tries to do it without referring to a counsellor.”

Participants differentiated between support from providers and broader support for women’s healthcare and described culture of support among healthcare providers that may not be reflected in wider social structures. Healthcare, though overall a supportive structure for women, did not decrease CBRC-seeking, suggesting policy impedes women’s access more than healthcare. Participants mentioned the expenses involved in CBRC-seeking, noting that individuals with financial instability may be unable to afford CBRC, forcing many couples to stop treatment. Women perceived MAR treatments to be less costly in other countries than in Italy, thus the increased interest in CBRC.

“From what I know of... the [healthcare] system is changing. At least from their doctors they get support. So, I don’t know if it is a widespread thing, but I would say there is a good support system. It is more the society [that’s the problem] ... for example, hospitals are starting to change their view of women’s healthcare access. But the public, they still need training in that. It is more of a prejudice. But the institutions are ahead of general.”

“[My friend] has contacted many different...private doctors, specialists. And then, they have guided her over different directions…because it was not legal in Italy to do this… so, she’s been traveling, it was on the east side of Europe, so she did [infertility treatment] there.”

“If you have an [infertility] problem and you cannot find a solution here, you have to go [abroad]. But to go out, you have to make the trip, pay the trip, find a place to stay during the therapy. So, it’s a lot of logistic things that you have to do.”

Those struggling with infertility also faced an emotional cost extending from the inability to fulfil societal norms. Participants implied CBRC-seeking magnified the already heavy emotional toll of infertility. Obtaining and undergoing treatment did not lessen the emotional impact of the process. Being around women who have succeeded in childbearing also brought about additional emotional pain; participants observed that infertile women often withdrew from friends and family. Women who sought CBRC appeared to have other reasons for withdrawing, including discomforting discussions after returning from abroad for treatment. Participant narratives illustrated the emotional effects of MAR and the need to seek CBRC are multifaceted.

“If you don’t have the services in your own country, going somewhere makes sense. But somehow it seems to be more shameful, doesn’t it? To have to go somewhere and do it.”

“And [besides] the emotional impact, I mean I’m sure if you arrive in another country to implant because you tried for perhaps four years and it doesn’t work, it hurts, it’s really painful. So, you also have to think about the trip, to be out of your home, it’s more difficult. And instead, if you could make it in Italy, at least you are in your home.”

## Discussion

Interviews offered in-depth insight into CBRC attitudes, behaviours, and experiences among a cohort of women living in Italy who had never engaged in CBRC. Social perspectives are critical to understanding the landscape of nuanced reproductive healthcare needs governed by policy and norms [37, 45, 46]. This is true of CBRC and infertility as these are couched within broad frameworks of religion, policy, identity and social acceptability. Prior research [22] demonstrated women faced obstacles to MAR that facilitated going beyond Italy for treatment and generated feelings of abandonment by the government. Though participants in our study had not experienced CBRC personally, women and couples within their networks had, and our participants highlighted multi-layered considerations of seeking CBRC. Because CBRC is a topic of conversation even among women who have not had these experiences, this suggests social norms and acceptability [15, 16, 47] of infertility treatments and CBRC are important considerations in healthcare choices. Understanding the broad perspectives regarding policy and social norms [15, 16] can better situate CBRC discussions within what is acceptable for Italians and can inform policy change or support for women and couples who desire treatments that are currently illegal or heavily regulated.

The Italian cultural value of the motherhood identity was significant in discussions surrounding infertility perspectives. Motherhood identity emphasizes the importance and respect placed on women who bear children in Italy [48], resulting in a paradox when considering infertility and CBRC. The importance of motherhood in Italian culture places societal pressure on women, leading to unrealistic motherhood expectations [48] and lowered quality of life [15, 16]. In cases of infertility, where achieving the goal of bearing children organically is not an option and restrictive policies in Italy make it difficult, women may seek alternative options (e.g., not becoming mothers, CBRC), which may be associated with social stigma or shaming. This pressure placed on women can make it difficult to fulfil the motherhood identity and may tie to the emotional costs and logistical barriers to CBRC. The desire to become a mother, coupled with restrictive policies, represent competing narratives of acceptable choices even among women who had never experienced infertility or CBRC. Thus, women’s voices, including personal experiences and social perspectives, are necessary for creating and implementing supportive policies reflecting the social norms and pressures women in Italy experience.

Despite the separation of church and state [5], the perception that religious influences pose barriers to women attempting to achieve their desired families remains. Thus, religious influence impacts the social climate and acceptability of MAR, making it necessary for people to travel to less religious or restrictive places to obtain MAR, which may result in monetary and emotional costs, including isolation and shame. The perception of trickle-down effects from religion into Italian policy was an accepted idea among participants, though they had never experienced CBRC. This may present a barrier in encouraging childbearing, as Italy’s notably low fertility rates have sparked efforts to encourage childbearing, such as Fertility Day campaigns [49]. Participants desired more supportive systems to have and raise healthy families; thus, the overarching perspective that religion enhances restrictive policies regarding childbearing opportunities should be addressed in policy, promotions, and campaigns that increase support for women to achieve their family planning goals. Despite laws having changed in 2014, women were still in belief of older laws and regulations. Though participants described the Italian healthcare system as supporting MAR-seeking, compassionate care is limited within the current environment, enhancing women’s perspectives of shame. Therefore, women’s voices should be included in policy to understand and address emotional costs and perceived barriers and create a landscape that supports women’s choices when, whether, and how to have a family within Italy.

### Strengths and limitations

Interviews were conducted with women who were comfortable speaking conversational English, which may have limited perspectives and vocabulary, therefore, some insights may not have been adequately captured. Additionally, women in this sample had higher education levels and were employed, which is to be expected from women capable of interviewing in English, thus limiting generalizability to women who may differ demographically and geographically. Interviews may have differed due to the flexibility of a semi-structured interview guide. As this was part of a larger study, not all questions in the interview guide were focused on CBRC and infertility perceptions. Interviews only took place in Florence, limiting generalisability and applicability to other regions of Italy. Despite these limitations, this study allowed us to explore the effects of societal and cultural subjectivism related to CBRC among Italian women. One strength was that the interview guide was reviewed by experts and in-country professionals, ensuring interview question quality and cultural relevance. An additional strength was conducting 30 interviews, which allowed for rich insights into myriad lived experiences and perspectives. Additionally, as part of a larger study on women’s health, CBRC these perspectives were situated in narratives of other related reproductive health conditions, which provided context and rich thought throughout the interview. However, no women in this study had experience with CBRC, which should be explored in a future study among a sample of women who have sought MAR through CBRC.

### Implications for health professionals and policymakers

Policymakers and practitioners should address social and cultural perceptions, increase access to safe and effective local care, and empower women in their family planning decisions. In particular, healthcare professionals should discuss the options available for those interested in building a family but who may be struggling with infertility. These should include in-country and CBRC options, so women and couples can be fully informed about the benefits and barriers of each. Furthermore, given the emotional toll that infertility and CBRC have on women, specifically, healthcare professionals should connect those experiencing infertility with mental health resources. Health promotion scholars should utilize social norms, like those discussed in this cohort of women who had not experienced CBRC, to identify barriers to seeking care and discussing infertility and CBRC with close social support systems. By identifying these barriers, they can craft social norming campaigns that reduce the stigma and isolation many women perceive occurring among those experiencing infertility of engaging in CBRC, helping to shift cultural perceptions. Women and couples may, in turn, feel more supported when considering having a family. Finally, policymakers should incorporate social perceptions of CBRC and infertility to lend constituency support for crafting MAR policies that expand the options available for individuals desiring to have a family. This can demonstrate the need for less restrictive policies, and empower women and couples in their childbearing goals.

### Future research

Future research should explore social opinions about CBRC, including MAR, among single and partnered women with these experiences. Further, to better understand women’s CBRC experiences, inclusion of women’s families and friends may be fruitful to better situate these within the social framework of women’s lives. A focus group methodology conducted in Italian and English may assist in gathering collective perceptions and interpretations, which can further elucidate the social climate surrounding CBRC. Additionally, future research should develop and test social norm campaigns aimed at reducing barriers, like stigma and motherhood identity failure associated with CBRC and infertility, to identify effective opportunities to empower women and couples in achieving their family planning goals. Finally, scholars should explore the perceptions of policymakers on CBRC, including those involved in past and current legislation, to demonstrate facilitators and gaps in creating supportive MAR policies.

## Conclusion

Findings offered insight into CBRC perceptions and intentions, presenting a deeper understanding of the existing family planning discourse among reproductive-aged women. This may allow policymakers and practitioners to address social and cultural perceptions, increase access to safe and effective local care, and empower women in their family planning decisions.

## Data Availability

The dataset used and analysed for the current study are available from the corresponding author upon reasonable request.
